# Prophages in *Salmonella enterica*: a driving force in reshaping the genome and physiology of their bacterial host?

**DOI:** 10.1111/mmi.14167

**Published:** 2018-12-25

**Authors:** Astrid Wahl, Aurélia Battesti, Mireille Ansaldi

**Affiliations:** ^1^ Laboratoire de Chimie Bactérienne, UMR7283, Institut de Microbiologie de la Méditerranée Centre National de la Recherche Scientifique, Aix‐Marseille Université Marseille France

## Abstract

Thanks to the exponentially increasing number of publicly available bacterial genome sequences, one can now estimate the important contribution of integrated viral sequences to the diversity of bacterial genomes. Indeed, temperate bacteriophages are able to stably integrate the genome of their host through site‐specific recombination and transmit vertically to the host siblings. Lysogenic conversion has been long acknowledged to provide additional functions to the host, and particularly to bacterial pathogen genomes where prophages contribute important virulence factors. This review aims particularly at highlighting the current knowledge and questions about lysogeny in *Salmonella* genomes where functional prophages are abundant, and where genetic interactions between host and prophages are of particular importance for human health considerations.

## Graphical abstract

*Salmonella enterica *genomes, as most other bacterial species, host multiple prophages whose genes can represent up to 5% of the genome content. In this review, we aim at putting into light the multiple genetic interactions that can be weaved between the host and its prophages and between prophages themselves within the host.

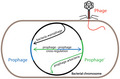

**Glossary**

Defective: a defective prophage has lost part of its genome and can no longer produce viral particles, but may still be able to excise from the host chromosome.

Functional: a prophage is considered functional if it can resume a lytic cycle and re‐infect naive cells.

Lysogeny: upon lysogenic infection by a temperate phage its genome is stably maintained as a prophage (most of the time integrated into the chromosome) in the host bacterium and vertically replicates with it. No progeny is produced until a lytic cycle resumes.

Lysogenic conversion: describes the phenotypic changes bacteria undergo upon infection by a temperate phage going through lysogeny.

Morons (“more on”): genes regulated independently from the rest of the prophage and conferring a fitness advantage under specific environmental conditions.

Polylysogeny: the hosting of multiple prophages in a single host genome.

Temperate bacteriophage: a phage that can multiply either lytically or through lysogeny.

Prophage/provirus: inherited form of a temperate bacteriophage; dormant form of the viral genome which replicates with the bacterial host genome.

Pseudolysogeny: a phage–host cell interaction in which the phage genome does not integrate into the host’s and forms an episomal form that can be still transmitted vertically. Pseudolysogeny applies to virulent and temperate phages and does not lead to the usual outcome lysis or lysogeny.

Pseudogenization: a gene becomes a pseudogene by accumulating mutations that hinder its correct transcription and/or translation.

Virulent or strictly lytic bacteriophage: a phage that multiplies exclusively through a lytic cycle.

## Introduction

Bacteriophages are recognized as the most abundant biological entities on earth, participating to numerous biological cycles and constantly reshaping bacterial communities (Suttle, 2007; Brussaard *et al*., 2008). In all environments they outcompete the number of available hosts by one to several log10. Moreover, due to their propensity to lysogenize, i.e. become quiescent proviruses, temperate phages are recognized as essential drivers of bacterial genomes’ evolution (Roux *et al*., 2015a; 2015b; Casjens and Grose, 2016) (some detailed definitions can be found in the glossary).

This review aims at highlighting the contribution of prophage genes to the host physiology. To date, a lot of emphasis has been put on the identification and characterization of phage‐encoded virulence factors in various pathogenic bacteria (Brüssow *et al*., 2004; Dearborn and Dokland, 2012; Rabinovich *et al*., 2012; Busby *et al*., 2012; Fortier and Sekulovic, 2013; Davies *et al*., 2016; Kraushaar *et al*., 2017). However, as it becomes clearer that bacterial genomes contain large amounts of DNA from (pro)phage origin, we want to stress that these horizontally acquired genes are important contributors to the genomes evolution and provide discrete adaptive physiological contributions such as increasing fitness under certain environmental conditions or providing non‐obvious metabolic or signaling functions (D’Ari and Casadesús, 1998). We chose to focus on *Salmonella enterica* prophages for the following reasons: (i) it is a widespread enterobacteria displaying a broad host range, frequently carried by wild and domestic birds as well as rodents, and an animal and human pathogen, (ii) host‐prophages interactions have been studied for many years and still lead to amazing pieces of work encompassing many topics, such as host‐phage interactions, virulence, ecology and genome evolution.

## Prophage abundance and integration sites in *S. enterica* genomes

The first prophages in *Salmonella* species have been identified in 1950, just before transduction has been discovered (Boyd, 1950; Zinder and Lederberg, 1952). Since the 1990s, the Bossi group has been a pioneer in *S. enterica* prophage research, which highlighted the diversity of the prophage repertoire of various strains (Figueroa‐Bossi *et al*., 1997; 2001; Figueroa‐Bossi and Bossi, 1999; Bossi *et al*., 2003). Since then many more prophages have been identified, every time a set of new *Salmonella* genomes is sequenced. Dormant prophages are transmitted vertically along with bacterial cell division and can be induced under stressful conditions, such as DNA damages or in animal guts (Kim *et al*., 2014). They can also undergo spontaneous induction, which can increase the fitness of a given strain whenever in competition when entering a new niche (Bossi *et al.,* 2003).

*Salmonella enterica* genomes also carry defective prophages that are no longer able to form infectious particles, meanwhile being present – and perhaps maintained – in the host chromosome (Casjens, 2003; Bobay *et al*., 2013). Different events can lead to prophage degradation including large genomic reduction, targeting by insertion sequences (IS) as well as point mutations (Bobay *et al*., 2014). However, when prophages are not too degraded it is possible to “resuscitate” them into fully functional prophages, meaning inducible and able to form infectious particles. Such reactivations of defective prophages involve either a temporal complementation by an infecting phage that provides the missing function, or a recombination event that allows a permanent complementation (Figueroa‐Bossi and Bossi, 2004; De Paepe *et al*., 2014). The recombination events driven either by the host homologous recombinases or phage‐encoded recombinases inside the host cells are causing pervasive mosaicism in phage genomes (Lopes *et al*., 2010; De Paepe *et al*., 2014; Menouni *et al*., 2015). The best known and long studied temperate phage infecting *S. enterica* is P22 (the λ equivalent paradigm in *S. enterica*) that was a key model for transduction discovery (Boyd, 1950; Zinder and Lederberg, 1952). However, P22 itself is not a common prophage in *S. enterica* genomes that contain in average 5.29 prophages representing around 3.52% of the total gene content and close to 30% of the accessory genome (based on 21 *S. enterica* genomes analyzed) (Bobay *et al*., 2013). In other words, these numbers show that polylysogeny, i.e. the hosting of multiple prophages by a single genome, is a very frequent event. Another striking point is that integration sites are highly conserved between the two closely related *Escherichia coli* and *S. enterica* species and even beyond (Bobay *et al*., 2013; Oliveira *et al*., 2017). Among favored integration sites are found all categories of non‐translated RNA genes such as sRNA, tmRNA and tRNA, the latter being the most frequently targeted (Bobay *et al*., 2013). Other sites in the chromosome may be targeted as well, such as intergenic regions, while integration within protein‐encoding genes is much less frequent. Even when integrating at the 3′ end of genes, the site‐specific reaction involved in the integration process leads to the reconstitution of the targeted genes since the equivalent portion of the gene is provided on the phage genome, without affecting their function or expression (Argos *et al*., 1986). Alternatively, a prophage may disrupt a gene and therefore a cellular function. However, when the prophage excises, the interrupted gene can be reconstituted and the host regains the lost function, a process called phage‐driven regulatory switch or active lysogeny (Feiner *et al*., 2015). However, no such a switch has been experimentally described so far in *S. enterica* genomes.

The quasi‐weekly release of new draft genomes from *S.* *enterica* prevents an accurate description of the prophage content as prophage description and annotation are not so obvious, even though facilitated by various softwares (Clokie and Kropinski, 2009). Indeed, the presence of multiple contigs may hinder the correct description of prophages as they frequently co‐localize with contig borders, impairing correct genome assembly and are sometimes interrupted by insertion sequences (IS). As a result, prophage predictions need to undergo expert manual curation.

A recent study based on public health surveillance in the UK highlighted that *S. enterica* Typhimurium causing invasive non‐typhoidal salmonellosis in Africa carried a specific prophage as well as antibiotic resistance genes that are not found in the UK version of this lineage (Kintz *et al*., 2015; Owen *et al*., 2017; Ashton *et al*., 2017). As a consequence stably integrated prophages are useful tools as epidemiology markers in addition to CRISPR‐Cas typing. However, as the latter were found to be poorly active and show a very slow spacer turnover, such typing should be restricted to anciently diverged strains (Touchon and Rocha, 2010).

## Lysogenic conversion

The notion of lysogenic conversion, meaning the propensity of temperate phage undergoing lysogeny to contribute to the host physiology has been described and admitted for numerous years. However, a strong bias is observed in the literature toward lysogenic conversion aspects that contribute the host virulence. As an example, the Gifsy‐2 encoded superoxide dismutase SodC that obviously contribute to the establishment of *Salmonella* cells into the macrophage (Figueroa‐Bossi and Bossi, 1999). Needless to say that *S. enterica* is an organism of choice for such contribution examples. However, one must consider that more subtle contributions do exist and pave the way for multiple interactions with the host genome as well as with the eukaryotic cells targeted by *S. enterica* or the microbiome encountered by the pathogen during its infectious journey in animals.

### Prophage induction and prophage gene expression under lysogenic conditions

**Figure 1:**
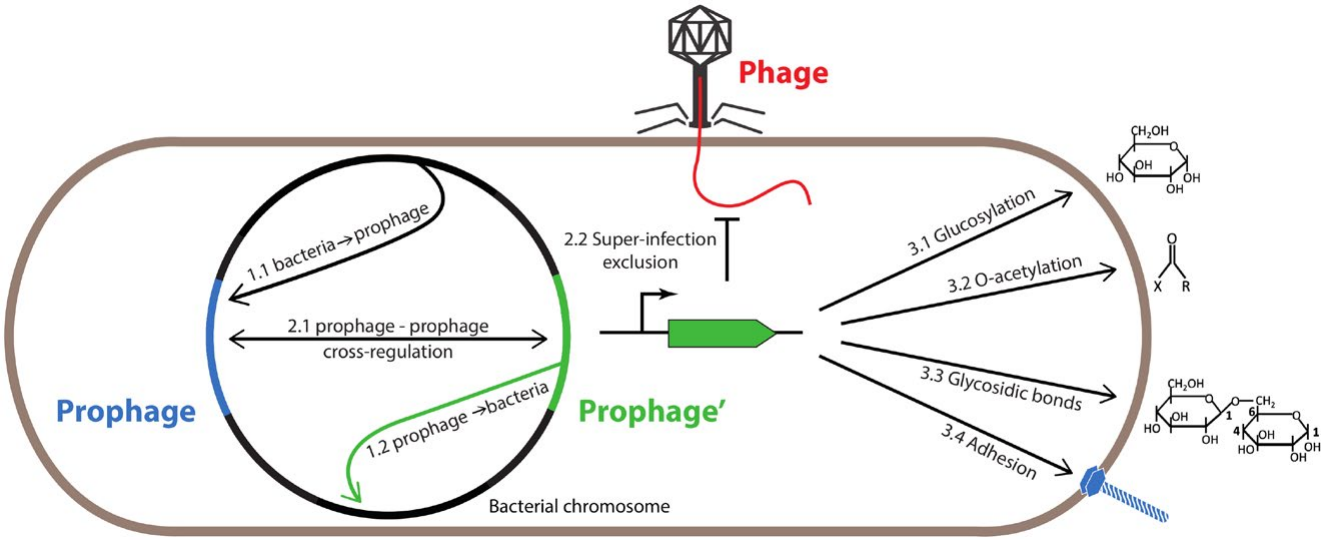
Multiple interactions between prophages and *Salmonella* hosts: The multiple host‐prophage and prophage‐prophage interactions depicted in the text are illustrated. For more details see Table 1.

As in *Escherichia coli*, the repressor model is widespread in *Salmonella*’s Lambdoid prophages (Sauer *et al*., 1981; Campbell, 1994; Whipple *et al*., 1998). However, a striking and widely conserved feature is the involvement of antirepressor proteins in prophage induction. If most *S. enterica* prophages are induced by the activation of the SOS response due to DNA damaging factors (mitomycine C, UV or H_2_O_2_), the cleavage of the repressor is not the major outcome of the induction system. Indeed, it was shown for several *S. enterica* prophages that upon SOS response induction and LexA self‐cleavage, an antirepressor protein (Ant), homologous to the Tum one in phage 186, is being made that inhibits the lytic repressor through protein‐protein interaction (Shearwin *et al*., 1998; Lemire *et al*., 2011; Kim and Ryu, 2013). P22 also encodes such an antirepressor whose expression is negatively controlled by the Mnt repressor. Nevertheless, in this case, an *ant *mutant remains SOS inducible (Botstein *et al*., 1975; Levine *et al*., 1975). Interestingly, antirepressors are responsible for prophage induction crosstalk: a given prophage‐encoded antirepressor was shown to counteract the action of a repressor from another prophage (Lemire *et al*., 2011) (Fig. 1 and Table 1 subitem 2.1). This prophage crosstalk has probably a role in prophage dissemination, as non‐coordinated prophages could be lost upon massive host cell lysis provoked by a neighboring prophage undergoing induction. In contrast, a partial cell lysis is often provoked by spontaneous or uncompleted induction, which allows a non‐induced prophage to remain in the bacterial population.

**Table 1:** Host‐prophage and prophage‐prophage interactions in *S. enterica*.


**1. Interactions bacteria – prophage and prophage – bacteria**
*1.1 Host factors*
Phase variation: Dam in conjunction with other regulator, such as OxyR H‐NS (silencing of exogenic DNA as heterodimers with proteins from Hha family) InvF (transcriptional regulator from AraC/ XylS family; *sopE*) SsrAB (two‐component system; *gogB*, *sseI*, *stm2239*)
*1.2 Prophage* *factors*
ΦW104 (*ryeA*, *ryeB*) Gifsy1 (AntQ forms complex with bacterial RNA polymerase)
**2. Interactions prophage** **– (pro)phage**
*2.1 Cross‐regulation*
Repressor – Antirepressor (Fels‐2 and Gifsy prophages)
*2.2 Super‐infection exclusion*
SieA and SieB (P22) Repressor C2 in cytoplasm (phage carrier state of P22)
**3. Surface features encoded by prophages** **and phage remnants**
*3.1 Glucosylation*
GtrABCa (phage remnant) GtrABC^P22^ (P22)
*3.2 O‐acetylation*
OafA (phage remnant) Protein similar to acyltransferase 3 (SPC‐P1 prophage) GtrABC^BTP1^ (BTP1; GtrC containing an acyltransferase domain)
*3.3 Glycosidic* *bonds*
phage beta polymerase changes to β‐1,6 glycosidic linkage (ε15; new phage host range: ε34)
*3.4 Adhesion*
tail‐like protein STM2699 (Fels‐2)

A number of inducing conditions or chemicals have been described that induce prophages in *Salmonella* through the SOS‐response activation. However, induction driven by some of these should lead to preoccupation in the context of public health risks. Indeed, it has been shown recently that some prophages are induced by antibiotics widely used in agriculture, such as fluoroquinolones (Bearson and Brunelle, 2015) and carbadox (Bearson *et al*., 2014). As a result, these antibiotics that are most probably present in our daily food intake may have thus consequences on prophage induction in our gut. Also relevant to the physiology of *Salmonella* could be the induction by bile salts, which are encountered by this bacterium in its natural ecological niche (Hernández *et al*., 2012). In all cases, little is known about the *in vivo* consequences of these treatments. However, one should consider the demonstration of *in vivo* transfer of prophage‐encoded virulence genes at loci of inflammation (Diard *et al*., 2017), which raises the question of the consequences of inducing antibiotics on the transmission of prophage‐encoded genes.

A commonly admitted view is that prophage genes are under control either by the powerful phage repressor or by host factors that tend to limit the negative effects of horizontal gene acquisition. In the first case, thanks to the seminal work of Jacob and Monod “The operon model” (Lewis, 2011), the lysogeny repressor is known to control the expression of the lytic promoters (P_L_ and P_R_) in the absence of induction. Host‐controlled expression of exogenic, and prophage genes in particular, has been described in several bacterial models (Navarre *et al*., 2006; Cardinale *et al*., 2008). However, it is in *S. enterica* that the role of the nucleoid associated protein H‐NS has been acknowledged as a genome sentinel (see below) (Navarre *et al*., 2006; Dorman, 2007; Navarre *et al*., 2007; Ali *et al*., 2013). Although there is no doubt about the extensive repression of prophage genes involved in lytic functions, we argue that it may have occulted the expression of those genes involved in lysogenic conversion and for which no obvious function in metabolic or pathogenic pathways have been identified. In order to find more of these genes whose expression remains significant under lysogenic conditions, one can look at the impressive amount of transcriptomic data available in the public databases and have a search in prophage‐related regions. One such very helpful database dedicated to *S. enterica* is the SalCom compendium developed by and hosted in Jay Hinton’s lab (Kröger *et al*., 2013; Colgan *et al*., 2016) (http://bioinf.gen.tcd.ie/cgi-bin/salcom.pl?_HL).

### Contributions to phage resistance and physiology

Being extremely abundant on earth while presenting a gene content very different from that currently available in the genomic databases, bacteriophage genomes are believed to contribute extensively to the genetic “dark matter” (Rinke *et al*., 2013; Roux *et al*., 2015b; Nadeem and Wahl, 2017). Indeed, even when focusing on a single bacterial host the gene repertoire carried by its infecting phages is highly diverse (Hatfull *et al*., 2010; Cresawn *et al*., 2011; Hatfull, 2015), and a large proportion of these genes remain annotated with unknown functions. A number of contributions to the host physiology might also beneficiate to the prophages themselves; among them are those preventing phage superinfection and/or recognition by the immune system from mammals.

#### LPS modifications

One particularity of phages infecting *Salmonella* strains is that they often recognize the lipopolysaccharide (LPS) or endotoxin consisting of a lipid and a polysaccharide composed of O‐antigen, as receptor as opposed to *E. coli* bacteriophages that often target outer membrane proteins (Bertozzi Silva *et al*., 2016). LPS contributes to the structural integrity and negative charge of the outer membrane of Gram‐negative bacteria and protects the membrane from certain kinds of chemicals, such as anionic bile salts or lipophilic antibiotics. Interestingly, the cognate *Salmonella* prophages frequently carry genes encoding proteins involved in O‐antigen modifications (Fig. 1 and Table 1 subitem 3) (Duerr *et al*., 2009; Broadbent *et al*., 2010; Andres *et al*., 2013; Davies *et al*., 2013; Sun *et al*., 2014; Cota *et al*., 2015; Kintz *et al*., 2015).

One of the most prominent examples is bacteriophage P22 of *S. enterica* which recognizes O‐antigen polysaccharides with its tailspike protein and when integrated as a lysogen in the bacterial chromosome provides itself a *gtrABC* operon for O‐antigen glucosylation (Andres *et al*., 2010; 2013). Up to four *gtr* operons encoded on different prophages and responsible for distinct modifications can be found within one bacterial genome (Broadbent *et al*., 2010). The *gtrA* and *gtrB* genes encode conserved membrane proteins, the bactoprenol‐linked glucosyl translocase or “flippase” and the bactoprenol glucosyl transferase respectively. It is the third gene, *gtrC*, in the cluster that is variable and confers specificity since it encodes the glucosyl transferase attaching a glucose group at a distinct O‐antigen position. These *gtr* operons are regulated according to phase variation (see the “Negative host‐control of prophage genes” section). LPS is a potent activator of immune cells and therefore these temporarily changing surface modifications can shade the bacteria vis‐à‐vis the eukaryotic immune system (Duerr *et al*., 2009). Besides glucosylation, another possible LPS O‐antigen modification is acetylation. An example is the gene *oafA* which is located on a phage remnant (at ~2.33 Mb on the *S. enterica* 4/74 chromosome) and codes for an integral membrane transacylase conferring the specific serotype 05 (Slauch *et al*., 1996). More recently, a gene coding for a protein similar to the acyltransferase 3 of *Pseudomonas syringae py. Syringae* B728a has been identified on prophage SPC‐P1, which was associated to increased virulence in *S. paratyphi* C (Zou *et al*., 2010). Another intriguing example is the *S. enterica* serovar Anatum specific phage ε15 which modulates glycosidic linkage of O‐antigen by blocking the host α polymerase and producing its own β polymerase (changes from α‐1,6 to β‐1,6 glycosidic linkage). This modification restricts super‐infection by ε15 itself and in turn allows infection by phage ε34 (Kropinski *et al*., 2007).

All these genetic features responsible for O‐antigen modifications and prophage‐encoded factors without being “real” virulence factors, may affect the resistance to the intestinal environment as well as to sur‐infecting phages, and thus in sum the fitness and pathogenicity of the bacterial host.

#### Superinfection exclusion

Besides the LPS modifications, phages have acquired other ways to prevent super‐infection by themselves and other phages. As examples we will mention here the genes *sieA* and *sieB* and the so‐called phage carrier state of P22 (Fig. 1 and Table 1 subitem 2.2). The inner membrane protein SieA is seemingly responsible for blocking the phage DNA transfer across the membrane into the bacterial cytoplasm (Susskind *et al*., 1974; Susskind and Botstein, 1980). SieB, also encoded by *E. coli* phage λ, aborts the lytic development of other *Salmonella* phages ‐ such as P22‐like MG178 and MG40 ‐ by stopping RNA, DNA, and protein synthesis. P22 itself is not affected, since it produces an early uncharacterized escape factor (Susskind *et al*., 1974). Interestingly, both SieA and SieB inhibit infection of the same phages, including λ, although *Salmonella* is not a standard host for the latter (Ranade and Poteete, 1993).

Phage carrier cells were identified recently and are cells infected with P22 harboring an episomal form of P22 that is transmitted asymmetrically during division (Cenens *et al*., 2013a; 2013b). The daughter cell inheriting this episome enters lysogeny resulting in a chromosomally integrated prophage. The other daughter cell becomes P22‐free, but intriguingly stays resistant to P22 infection in a transient way. The immunity factors responsible for this resistance are GtrABC, SieA and the repressor C2, which have been constitutively produced by the phage carrier cell. The P22‐free daughter cells cytoplasmically inherit these immunity factors, which then dilute out upon subsequent cell divisions (Cenens *et al*., 2015). By conferring this temporary resistance to a bacterial subpopulation, phages might thus insure both vertical and horizontal transmission routes throughout an infected population while maintaining a bacterial population they can infect. This process, described as “host‐farming” by A. Aertsen, allows P22 to cultivate susceptible non‐immune cells as a prey reservoir. This is an example of how up‐to‐date single‐cell studies contribute to the field and also shows that even the extensively studied phages such as P22 still reserves surprising new features to uncover.

### Contribution of prophage gene products to virulence

This section will be mainly dedicated to recent or not yet reviewed examples of prophage‐encoded factors that are involved in *Salmonella *virulence (Boyd and Brüssow, 2002; Boyd, 2012; Boyd *et al*., 2012). *Salmonella* displays full panoply of virulence factors permitting to adhere to and infect eukaryotic cells and survive within them, in particular the hostile microenvironment of macrophages.

#### Adhesion

Shah et al*.* have identified a prophage gene, *gpE,* coding for a putative tail‐spike protein in SopEΦ in *S. enterica* LT2 that increased binding to epithelial cells (specifically *via* Spectrin1, an eukaryotic surface protein) and increased cell invasion (Shah *et al*., 2014; Fig. 1, Table 1 subitem 3.4). However, this prophage‐encoded gene was only expressed when bacteria were exposed to a cold stress before the infection assay. This emphasizes the importance of storage conditions for *Salmonella* contaminated food (mostly eggs and poultry) that are stored at cold and then reheated exposing bacteria to a chain of stresses, which may in turn induce prophages genes and finally, increase bacterial virulence.

#### Host entry, manipulation and intracellular survival (virulence factors per se)

One of the most impressive features of *S. enterica* virulence is the secretion of multiple effectors involved in virulence. The effectors are secreted *via* two different Type Three Secretion Systems (T3SS) encoded by the pathogenicity islands 1 and 2 (SPI1 and 2). SPI1 is activated when *S. enterica* is in contact with eukaryotic host cells, whereas SPI2 is expressed during the phagocytosis step (Kaur and Jain, 2012). Bacterial effectors are able to interfere and hijack the host signaling pathways. In addition, other SPI‐encoded factors exist that facilitate bacterial survival, among which so‐called anti‐virulence factors, which deletions curiously confer more virulence to the bacteria.

Among the SPI1 secreted effectors, SopE is one of the best studied effector proteins and its secretion results in actin cytoskeleton rearrangements and stimulates membrane ruffling, promoting bacterial entry into non‐phagocytotic cells such as epithelial cells (reviewed in (Ehrbar and Hardt, 2005)). Its encoding gene has been originally identified on the SopEΦ prophage in *S. enterica* SL1344, but the *sopE* gene and a constant flanking sequence, called the SopE‐cassette, is sporadically distributed in other lambdoid prophages of the Gifsy family among several *Salmonella* serovars, as well as on a P2‐like prophage in *S. typhi* (Mirold *et al*., 2001; Bachmann *et al*., 2014). The SopE‐cassette has most probably been transferred and integrated among these prophages by homologous recombination resulting in multiple *sopE* copies present in a single bacterial genome (Hoffmann *et al*., 2014). In the context of evolution, such a modular exchange mechanism could enhance effector protein diversity, since genes may duplicate and then potentially evolve to other functions. However, even the well studied SopE virulence factor may still not have unveiled all its functions. Indeed, recently SopE has been found to be not only produced and secreted for entering the eukaryotic cell, but also during the intracellular state where it seems to participate to the formation of the early *Salmonella*‐containing vacuole (SCV) (Vonaesch *et al*., 2014). The SCV is formed in order to create a replicative niche for the bacteria within the host cell. This double function of SopE during cell entry as well as during intracellular survival suggests that other prophage‐encoded effectors may have additional functions for host‐cell manipulation.

Gifsy1 prophage has been found to encode three genes involved in intra‐cellular survival: *gogB*, *sarA*, and *pagK2*. The first gene, *gogB*, codes for an anti‐inflammatory effector, which inhibits NFκB activation by interaction with host factors Skp1 and FBX022. It is thought that this anti‐inflammatory effect limits tissue damage during longer term infection, while short‐term inflammation enhances colonization in the intestine (Pilar *et al*., 2012).

The second gene, *sarA*, has been identified only very recently. SarA is mainly secreted by SPI2‐encoded T3SS, although there is also some translocation by SPI1 T3SS. It activates the eukaryotic transcription factor STAT3, which induces the transcription of Il‐10 as well as of other anti‐inflammatory factors. SarA is thus the first example of an effector that activates an anti‐inflammatory pathway in the eukaryotic host cell (Jaslow *et al*., 2018). PagK2 is secreted in outer membrane vesicles and contributes to intracellular survival in macrophages through an unknown mechanism (Yoon *et al*., 2011). The anti‐inflammatory effects of the T3SS effectors seem to be crucial at systemic sites later in infection when *S. enterica* must evade immunity and promote intracellular growth. Apparently, there is an evolutionary advantage to maintain *gogB* and *sarA* on the same prophage and the recent identification of a new prophage ST‐1974 in *S.* *enterica* Enteritidis supports this idea (D’Alessandro *et al*., 2018). In this case, the two genes coding for anti‐inflammatory functions, *gogB* and *ssek3*, are present on a single prophage. As mentioned above, *gogB* is encoded on Gifsy1 but can be found elsewhere on the chromosome (see below), while *sseK3* has been previously identified on prophage ST64B (Brown *et al*., 2011). So, it seems that recombination events, similar to the above‐mentioned modular exchange of the SopE‐cassette, have taken place between these prophages. Interestingly, both GogB and SseK3 act on the same anti‐inflammatory NFκB pathway. However, the SseK3 host targets remain to be identified (Yang *et al*., 2015).

#### Antivirulence

Some prophage‐encoded genes confer an intriguing phenotype termed anti‐virulence. The Gifsy2‐encoded *grvA* gene is such an anti‐virulence factor: in its absence, and in contrast to what one would expect with classical virulence genes, the bacterial host is *more* virulent than a wild type strain in competition assays in mice (Ho and Slauch, 2001). However, this phenotype is only observed when *sodCI*, a Gifsy2‐encoded superoxide dismutase, is present as well. Thus, it is hypothesized that in a wild type situation, GrvA decreases the pathogenicity of the host probably by affecting resistance to toxic oxygen species via SodCI through an unknown mechanism. Another peculiar example of a factor that can be a virulence or an anti‐virulence factor depending on the serovar type of its host, is *bstA* encoded on prophage BTP1. Indeed, it acts as virulence factor in *S. enterica* ST313, i.e. higher uptake in macrophages (Herrero‐Fresno *et al*., 2014), while it was described as an anti‐virulence factor (lower uptake) in *S. enterica* Dublin; however the molecular mechanisms underlying both phenotypes are not yet understood (Herrero‐Fresno *et al*., 2018). A potential reason for this difference may be that, similarly to GrvA, another virulence factor is affected by BstA and is present in only one serovar. This highlights the possibility of a different output of prophage genes depending of the pre‐existing bacterial regulatory networks. Currently, it is not fully understood why bacterial pathogens would possess these antivirulence genes and what the evolutionary advantage (for both the prophage and the host) might be. One may speculate that bacterial pathogens might evolve toward less virulence in order to ensure their own propagation by keeping the potential host in shape in a way resembling to the above described phage‐carrier state. The fact that these factors are prophage‐encoded might give an advantage to changing environmental niches of the mammal’s body, since prophages can be lost and acquired in only one recombination event in the gut and therefore provide a fast way of adaptation (Diard *et al*., 2017).

## Host‐prophage regulatory networks

As mentioned earlier, genes from phage origin represent a large part of *S. enterica* accessory genome. Some of these genes contribute to the host physiology and therefore need to be expressed at the right time and the right place. To this end, they become part of the bacterial regulatory network. How does acquisition of these new genes not disturb the normal bacterial functioning? How are they integrated into the host regulatory network? What potential benefit do they provide to the bacterial host? How can bacterial regulators modulate prophage behavior by modulating gene expression?

### Negative host‐control of prophage genes

Expression of new genes must not be detrimental for the bacterial host. Therefore, genes acquired by horizontal gene transfer (HGT) are generally first silenced before being integrated into the host regulatory network. The silencing of genes from foreign origin can, for example, occur *via* DNA modification or involve regulatory proteins that bind DNA to prevent transcription.

One of the most studied DNA modifications is responsible for an epigenetic regulation called phase variation and occurs only in a small fraction of the bacterial population. This regulation relies on the methylation of deoxyadenosines by the Dam methylase (Deoxyadenosine methyltransferase) (Casadesús, 2016). The Dam enzyme recognizes and specifically modifies the 5′‐GATC‐3′ sequences; when these sequences are localized in a promoter region, methylation events can block the binding of transcriptional regulators and consequently modify gene expression. DNA methylation is involved in the silencing of genes localized on the Gifsy1, Fels1 and ST64B prophages in *S. enterica *SL1344 (Balbontín *et al*., 2006). Strikingly, it negatively regulates most of the ST64B genes. This observation is in accordance with previous results showing that ST64B excision is inhibited by Dam regulation (Alonso *et al*., 2005). This has been attributed to the down regulation of two genes located on this prophage and coding for proteins involved in phage induction: the anti‐repressor Sb41 and the replication protein Sb42. The bacterial regulator involved in this regulation and hindered in its function by the Dam methylation has not been identified to date. While the Dam‐regulation observed for genes located on Gifsy1 and Fels1 prophages does not affect their excision, SopEΦ prophage excision is favored by Dam methylation. However, the transcriptional regulator as well as the target genes responsible for this phenotype has not been described (Alonso *et al*., 2005).

Epigenetic regulation is also involved in the regulation of O‐antigen glucosylation (see LPS modification section). Indeed, under lysogenic conditions expression of the P22 encoded *gtrABC* operon is regulated by Dam methylation and the bacterial regulator OxyR (Broadbent *et al*., 2010; Davies *et al*., 2013). The region upstream of the *gtr* operon contains several OxyR binding sites as well as several methylation sites. Depending on the methylation state, OxyR bind different sites and can act as an activator or a repressor of the system. OxyR binding to one site decreases the methylation by Dam on this site and thus increases its own binding. But this works also the other way around: increased methylation results in reduced OxyR binding, which favors methylation, etc. This confers heritability of the expression state to the system and only a part of the population is in an “ON” state, leading to an heterogeneous population (Broadbent *et al*., 2010; García‐Pastor *et al*., 2018). This regulatory mechanism is thought to be conserved among the P22 temperate phage family and can prevent superinfection by the same or other phages that use similar O‐antigen co‐receptor during a limited time (Davies *et al*., 2013).

Another factor involved in the silencing of genes acquired by HGT, in addition to core genes, is the DNA binding protein H‐NS (Lucchini *et al*., 2006; Navarre *et al*., 2006). By preferentially binding to AT rich sequences, this protein can discriminate between self and non‐self. Interestingly, several studies suggest that H‐NS dependent regulation would involve different mechanisms for ancestral genes or genes acquired by HGT (Vivero *et al*., 2008; Baños *et al*., 2009). It has been suggested that ancestral genes would be regulated directly by H‐NS binding whereas the genes acquired by HGT would require the formation of heterodimers between H‐NS and proteins belonging to the Hha family. What characteristics of the promoter are required to favor the binding of homodimers or heterodimers are not known. In the same order of idea, H‐NS proteins encoded by conjugative plasmids have evolved to specifically regulate foreign genes (Baños *et al*., 2009; 2011). This could be due to structural differences between plasmid‐encoded or chromosomally encoded H‐NS leading to different affinity for promoter regions. Indeed, although the N‐ and C‐terminal domains are conserved, the linker region presents some variability that could be responsible for this differential regulation. All these observations concern genes acquired by HGT in general, including genes from phage origin. However, it has been noticed that the GC content of prophages in the reference strain of *S. enterica* LT2 is similar to the average GC content of the genome (Navarre *et al*., 2007). Thus, we can wonder if the conclusions made above really apply to genes from prophage origin. Studies focusing specifically on the regulation of these genes are missing so far and need to be performed to answer this question. Ongoing work in our lab suggests however that H‐NS regulates prophage genes that have not been identified by global approaches in *S. enterica* ST4/74 and that these regulations have consequences on prophage maintenance in the host chromosome (Wahl *et al*., unpublished).

### Positive host‐control of prophage genes

In addition to the negative regulation that we have just mentioned, some bacterial regulators also positively regulate genes from prophage origin. Surprisingly, if one looks at the different transcriptomic studies performed in *S. enterica* to define the targets of global regulator such as PhoP, SlyA, ArcA, FNR or RpoS, only a handful of prophage genes were identified (Navarre *et al*., 2005; Fink *et al*., 2007; Evans *et al*., 2011; Lévi‐Meyrueis *et al*., 2015). Furthermore, the molecular mechanism(s) leading to these regulations or the consequences on bacterial physiology are rarely looked at. Not surprisingly, what has been mostly studied is the regulation of genes coding for proteins involved in virulence and host colonization. These studies have shown that genes under the control of bacterial regulators are morons, which defines genes regulated independently from the rest of the prophage and conferring an advantage (fitness effect under specific conditions such as virulence) to the host. Among them, are several effectors proteins secreted by T3SS. As mentioned above, *S. enterica* possesses two T3SS encoded by the pathogenicity island 1 and 2 (SPI1 and 2). Among the regulators known to control the expression of genes located on the SPI are the InvF transcriptional regulator belonging to the AraC/XylS family for SPI1, itself regulated by the master regulator HilD, and the SsrAB two‐component system for SPI2. Although both regulators were initially thought to be only dedicated to the regulation of genes located on SPI1 and 2, they also regulate expression of prophage‐encoded effectors. Indeed, InvF regulates effectors from prophage origin secreted by SPI1, whereas SsrAB controls effectors that are SPI2‐dependent.

For example, SopE is a virulence factor encoded on the SopEΦ prophage in *S. enterica* SL1344 and secreted by the SPI1 system. Consequently, *sopE* expression is regulated by InvF, in association with the chaperon protein SicA (Darwin and Miller, 2000). The role of SicA is probably indirect, by stabilizing or allowing the function of a so far unidentified transcriptional regulator involved in *sopE* regulation (Tucker and Galán, 2000).

Other prophage‐encoded effectors are secreted by SPI2, and as a consequence, their expression depends on the SsrAB two‐component system. Among them, GogB is encoded by the first gene of the Gifsy1 prophage in *S. enterica* SL1344. Interestingly it has been shown that *gogB* expression is independent of Gifsy1 prophage factors since it can be transferred by itself in the enteropathogenic *E. coli* strain E2348/69, expressed from its own promoter and secreted *via* the T3SS of its new host. This shows that *gogB* can be integrated easily into the host regulatory network (Coombes *et al*., 2005). The GC content of *gogB* shows differences with the GC content of Gifsy1 suggesting that this gene has been recently acquired by the prophage. Moreover, *gogB* can be found outside of Gifsy1 and is not always prophage‐encoded, which further supports its transcriptional independence from the Gifsy1 prophage (Porwollik *et al*., 2002).

*sseI* is a gene located on the Gifsy2 prophage, encoding another T3SS effector. *sseI* expression is strongly activated by the direct binding of the phosphorylated form of SsrB in its promoter region (Worley *et al*., 2000; Feng *et al*., 2004). *sseI* expression is also regulated by the phosphorylated form of OmpR but it is not clear whether this regulation is direct or dependent on SsrB (Feng *et al*., 2004). Interestingly, the pseudogenization of *sseI* together with the higher expression of *pgtE*, encoding an outer membrane protein, allows *S. enterica* ST313 adaptation to cause systemic disease (Carden *et al*., 2017; Hammarlöf *et al*., 2018). The increase in *pgtE* transcription is due to a single SNP in its promoter region. Further studies are required to understand how these changes in gene expression modify *S. enterica* ST313 behavior (Hammarlöf *et al*., 2018).

SsrB also regulates genes in the phage remnant SPI12. Among the regulated genes STM2239 encodes a Q antiterminator protein that interacts with the RNA polymerase to facilitate the transcription of late promoters. The absence of STM2239 affects the fitness of the bacteria within the host. STM2239 allows the transcription of phage‐encoded genes but also of bacterial genes involved in metabolic pathways including ribose modification and transport, acetyl coenzyme A synthesis and recycling as well as galactose metabolism. None of these regulations have been characterized further, but it has been speculated that some of them may be important for *S. enterica* fitness within the host (Tomljenovic‐Berube *et al*., 2013).

### Phage‐controlled bacterial genes

Except for virulence, examples of bacterial processes under prophage control are scarce (Fig. 1 and Table 1 subitem 1.2). However, P22 offers a nice illustration of bacterial genes encoding proteins involved in metabolism and under the control of a regulator from phage origin. The *dgo* operon is involved in the uptake and metabolism of D‐galactonate, an important carbon source during intracellular proliferation. In *S. enterica* LT2 strain, expression of the *dgo* operon is derepressed in the presence of Pid, a protein encoded on a moron locus in P22 (Cenens *et al*., 2013a; 2013b). This regulation only occurs when P22 undergoes pseudolysogeny, suggesting the existence of a dedicated genetic program in this condition.

Phage‐dependent regulation can be conserved among closely related bacteria. It is the case for the *pckA* gene encoding a phosphoenolpyruvate carboxykinase required for gluconeogenesis. In *E. coli*, this gene is under the control of the CI repressor of the λ phage (Chen *et al*., 2005). Interestingly, *pckA* regulatory region is conserved among *Enterobacteriaceae* and contains sequences homologs to several phage operators, one of them being the binding site for the C2 repressor of P22. This shared regulation between several prophages could be part of an adaptive strategy to increase lysogens fitness by lowering their growth rate under glucose‐limited conditions (Chen *et al*., 2005).

Prophages often integrate into tRNA encoding genes. One counter example is given by phage ΦW104 that integrates the host chromosome at a locus encoding the RyeA and RyeB sRNA located on the opposite DNA strand in *S. enterica* DT104 (Balbontín *et al*., 2008). The attachment site for ΦW104 is within the 23 last base pairs of *ryeB* and corresponds to an internal site in *ryeA*. Therefore, ΦW104 lysogenization modifies the 5′ portion of *ryeA*, leading to a decreased transcription of *ryeA* and an increased transcription of *ryeB*. This transcriptional regulation has probable physiological consequences on the bacterial host, by modifying the expression of RyeA and RyeB mRNA targets.

Finally, another example of host‐gene regulation involves a gene located on the Gifsy1 prophage, *isrK* (Hershko‐Shalev *et al*., 2016). This gene encodes an sRNA and a long polycistronic mRNA comprising *isrK*, *orf45*, *anrP* and *isrJ* coding sequences. However, there is no translation observed from this mRNA unless IsrK sRNA is present. Indeed, IrsK sRNA binds next to the *orf45* ribosome binding site and facilitates the binding of the 30S ribosomal subunit to this site, leading to the translation of downstream sequences and the production of the AnrP protein. AnrP is an anti‐repressor activating the transcription of phage‐encoded genes. Among them, it activates the expression of the *antQ* gene coding for the AntQ anti‐terminator that interacts and forms a stable complex with RNA polymerase. This leads to an aberrant transcriptional elongation, DNA damage and ultimately cell death (Hershko‐Shalev *et al*., 2016).

All the above examples concern regulation of gene expression. However, phages can also influence bacterial physiology by other means. For example, the release of colicin 1b in *S. enterica *SL1344 depends on the lysis genes of the ST64B prophage (Nedialkova *et al*., 2015). Indeed, under specific conditions such as DNA damage or iron limitation, colicin 1b accumulates in the cell and needs the induction of ST64B lysis genes to be found in the extracellular medium. Interestingly, complex crosstalk between ST64B and other prophages present in that strain contributes to this regulation and need further characterization.

## Conclusions

What is striking whenever considering and comparing different *Salmonella* genomes is the diversity of the prophage content as well as the diverse relationships these prophages engage with the host strains. As stated before, we think that contributions to virulence have been more studied and highlighted up to now than regulatory and metabolic interactions between the *Salmonella* host and its prophages. This bias seems largely due to the prevalent role of *Salmonella* species in public health threats. In addition, contrary to some previous statements, phage genomes rarely contain antibiotic resistance genes, and if antibioresistance transfer can be sometimes attributed to phages, it is more likely due to generalized transduction rather than lysogeny (Enault *et al*., 2017). Nevertheless, a new category of self transferable plasmid‐phages could change this view as some of them carry ATB resistance genes (SSU5 super‐cluster) (Gilcrease and Casjens, 2018).

Apart from actual contributions to virulence, we foresee that many more interactions do exist, and, it seems that microbiologists have only just began to explore the expense of prophage–host interactions and their short and long‐term effects on bacterial metabolism and evolution. More interactions certainly remain to be discovered such as the up‐to‐now neglected carrier state and its implications on the host metabolism (Cenens *et al*., 2015).

In a context of multidrug resistance spreading and recurrent warnings from WHO and other health authorities, the use of bacteriophages as therapeutic agents is coming back to the scene, not only as potent antimicrobials by themselves but also as synergistic or complementary agents in combination with antibiotics (Kamal and Dennis, 2015; Abedon, 2018). However, even though only virulent (or strictly lytic) phages are considered for therapeutic usage, one must be aware of the possible interference of prophages whenever considering phages as a treatment. As described above, prophages are important contributors of serotype conversion, and particularly in *Salmonella* species. The literature becomes quite abundant regarding *Salmonella* phage quests, but little is known about the consequences of poly‐lysogeny, which can rapidly modify the bacterial surface, and therefore, the resistance to surinfecting phages on the efficacy of phage cocktails, particularly for those developed to treat swine and poultry or in the case of adjuvant in food processing (Wernicki *et al*., 2017). We suggest to systematically assessing the prophage content of the targeted strains to evaluate and adapt the composition of phage cocktails.

## References

[mmi14167-bib-0001] Abedon, S.T. (2018) Phage therapy: various perspectives on how to improve the art. Methods in Molecular Biology (Clifton, NJ), 1734, 113–127. 10.1007/978-1-4939-7604-1_11 29288451

[mmi14167-bib-0002] Ali, S.S. , Whitney, J.C. , Stevenson, J. , Robinson, H. , Howell, P.L. and Navarre, W.W. (2013) Structural insights into the regulation of foreign genes in *Salmonella* by the Hha/H‐NS complex. The Journal of Biological Chemistry, 288, 13356–13369. 23515315 10.1074/jbc.M113.455378 PMC3650374

[mmi14167-bib-0003] Alonso, A. , Pucciarelli, M.G. , Figueroa‐Bossi, N. and García‐del Portillo, F. (2005) Increased excision of the *Salmonella *prophage ST64B caused by a deficiency in Dam methylase. Journal of Bacteriology, 187, 7901–7911. 16291663 10.1128/JB.187.23.7901-7911.2005 PMC1291290

[mmi14167-bib-0004] Andres, D. , Gohlke, U. , Broeker, N.K. , Schulze, S. , Rabsch, W. , Heinemann, U. , et al. (2013) An essential serotype recognition pocket on phage P22 tailspike protein forces *Salmonella* *enterica* serovar Paratyphi A O‐antigen fragments to bind as nonsolution conformers. Glycobiology, 23, 486–494. 23292517 10.1093/glycob/cws224

[mmi14167-bib-0005] Andres, D. , Hanke, C. , Baxa, U. , Seul, A. , Barbirz, S. and Seckler, R. (2010) Tailspike interactions with lipopolysaccharide effect DNA ejection from phage P22 particles in vitro. The Journal of Biological Chemistry, 285, 36768–36775. 20817910 10.1074/jbc.M110.169003 PMC2978605

[mmi14167-bib-0006] Argos, P. , Landy, A. , Abremski, K. , Egan, J.B. , Haggard‐Ljungquist, E. , Hoess, R.H. *et al* (1986) The integrase family of site‐specific recombinases: regional similarities and global diversity. The EMBO Journal, 5, 433–440. 3011407 10.1002/j.1460-2075.1986.tb04229.x PMC1166749

[mmi14167-bib-0007] Ashton, P.M. , Owen, S.V. , Kaindama, L. , Rowe, W.P.M. , Lane, C.R. , Larkin, L. *et al* (2017) Public health surveillance in the UK revolutionises our understanding of the invasive *Salmonella *Typhimurium epidemic in Africa. Genome Medicine, 9, 92. 29084588 10.1186/s13073-017-0480-7 PMC5663059

[mmi14167-bib-0008] Bachmann, N.L. , Petty, N.K. , Ben Zakour, N.L. , Szubert, J.M. , Savill, J. and Beatson, S.A. (2014) Genome analysis and CRISPR typing of *Salmonella enterica* serovar Virchow. BMC Genomics, 15, 389. 24885207 10.1186/1471-2164-15-389 PMC4042001

[mmi14167-bib-0009] Balbontín, R. , Figueroa‐Bossi, N. , Casadesús, J. and Bossi, L. (2008) Insertion hot spot for horizontally acquired DNA within a bidirectional small‐RNA locus in *Salmonella enterica* . Journal of Bacteriology, 190, 4075–4078. 18390661 10.1128/JB.00220-08 PMC2395037

[mmi14167-bib-0010] Balbontín, R. , Rowley, G. , Pucciarelli, M.G. , López‐Garrido, J. , Wormstone, Y. , Lucchini, S. *et al* (2006) DNA adenine methylation regulates virulence gene expression in *Salmonella enterica* serovar Typhimurium. Journal of Bacteriology, 188, 8160–8168. 16997949 10.1128/JB.00847-06 PMC1698197

[mmi14167-bib-0011] Baños, R.C. , Aznar, S. , Madrid, C. and Juárez, A. (2011) Differential functional properties of chromosomal‐ and plasmid‐encoded H‐NS proteins. Research in Microbiology, 162, 382–385. 21320594 10.1016/j.resmic.2011.02.003

[mmi14167-bib-0012] Baños, R.C. , Vivero, A. , Aznar, S. , García, J. , Pons, M. , Madrid, C. *et al* (2009) Differential regulation of horizontally acquired and core genome genes by the bacterial modulator H‐NS. PLoS Genetics, 5, e1000513. 19521501 10.1371/journal.pgen.1000513 PMC2686267

[mmi14167-bib-0013] Bearson, B.L. , Allen, H.K. , Brunelle, B.W. , Lee, I.S. , Casjens, S.R. and Stanton, T.B. (2014) The agricultural antibiotic carbadox induces phage‐mediated gene transfer in *Salmonella* . Frontiers in Microbiology, 5, 52. 24575089 10.3389/fmicb.2014.00052 PMC3920066

[mmi14167-bib-0014] Bearson, B.L. and Brunelle, B.W. (2015) Fluoroquinolone induction of phage‐mediated gene transfer in multidrug‐resistant *Salmonella* . International Journal of Antimicrobial Agents, 46, 201–204. 26078016 10.1016/j.ijantimicag.2015.04.008

[mmi14167-bib-0015] Bertozzi Silva, J. , Storms, Z. and Sauvageau, D. (2016) Host receptors for bacteriophage adsorption. FEMS Microbiology Letters, 363. 10.1093/femsle/fnw002 26755501

[mmi14167-bib-0016] Bobay, L.‐M. , Rocha, E.P.C. and Touchon, M. (2013) The adaptation of temperate bacteriophages to their host genomes. Molecular Biology and Evolution, 30, 737–751. 23243039 10.1093/molbev/mss279 PMC3603311

[mmi14167-bib-0017] Bobay, L.‐M. , Touchon, M. and Rocha, E.P.C. (2014) Pervasive domestication of defective prophages by bacteria. Proceedings of the National Academy of Sciences, 111, 12127–12132. 10.1073/pnas.1405336111 PMC4143005 25092302

[mmi14167-bib-0018] Bossi, L. , Fuentes, J.A. , Mora, G. and Figueroa‐Bossi, N. (2003) Prophage contribution to bacterial population dynamics. Journal of Bacteriology, 185, 6467–6471. 14563883 10.1128/JB.185.21.6467-6471.2003 PMC219396

[mmi14167-bib-0019] Botstein, K. , Lew, K.K. , Jarvik, V. and Swanson, C.A. (1975) Role of antirepressor in the bipartite control of repression and immunity by bacteriophage P22. Journal of Molecular Biology, 91, 439–462. 1097697 10.1016/0022-2836(75)90271-5

[mmi14167-bib-0020] Boyd, E.F. (2012) Bacteriophage‐encoded bacterial virulence factors and phage‐pathogenicity island interactions. Advances in Virus Research, 82, 91–118. 22420852 10.1016/B978-0-12-394621-8.00014-5

[mmi14167-bib-0021] Boyd, E.F. and Brüssow, H. (2002) Common themes among bacteriophage‐encoded virulence factors and diversity among the bacteriophages involved. Trends in Microbiology, 10, 521–529. 12419617 10.1016/s0966-842x(02)02459-9

[mmi14167-bib-0022] Boyd, E.F. , Carpenter, M.R. and Chowdhury, N. (2012) Mobile effector proteins on phage genomes. Bacteriophage, 2, 139–148. 23275865 10.4161/bact.21658 PMC3530523

[mmi14167-bib-0023] Boyd, J.S.K. (1950) The symbiotic bacteriophages of *Salmonella *typhimurium. The Journal of Pathology and Bacteriology, 62, 501–517. 14804230 10.1002/path.1700620402

[mmi14167-bib-0024] Broadbent, S.E. , Davies, M.R. and Van Der Woude, M.W. (2010) Phase variation controls expression of *Salmonella* lipopolysaccharide modification genes by a DNA methylation‐dependent mechanism. Molecular Microbiology, 77, 337–353. 20487280 10.1111/j.1365-2958.2010.07203.x PMC2909390

[mmi14167-bib-0025] Brown, N.F. , Coombes, B.K. , Bishop, J.L. , Wickham, M.E. , Lowden, M.J. , Gal‐Mor, O. *et al* (2011) *Salmonella* phage ST64B encodes a member of the SseK/NleB effector family. PLoS One, 6, e17824. 21445262 10.1371/journal.pone.0017824 PMC3060822

[mmi14167-bib-0026] Brussaard, C.P.D. , Wilhelm, S.W. , Thingstad, F. , Weinbauer, M.G. , Bratbak, G. , Heldal, M. *et al* (2008) Global‐scale processes with a nanoscale drive: the role of marine viruses. The ISME Journal, 2, 575–578. 18385772 10.1038/ismej.2008.31

[mmi14167-bib-0027] Brüssow, H. , Canchaya, C. and Hardt, W.‐D. (2004) Phages and the evolution of bacterial pathogens: from genomic rearrangements to lysogenic conversion. Microbiology and Molecular Biology Reviews, 68, 560–602. 15353570 10.1128/MMBR.68.3.560-602.2004 PMC515249

[mmi14167-bib-0028] Busby, B. , Kristensen, D.M. and Koonin, E.V. (2012) Contribution of phage‐derived genomic islands to the virulence of facultative bacterial pathogens. Environmental Microbiology, 15, 307–312. 23035931 10.1111/j.1462-2920.2012.02886.x PMC5866053

[mmi14167-bib-0029] Campbell, A. (1994) Comparative molecular biology of lambdoid phages. Annual Review of Microbiology, 48, 193–222. 10.1146/annurev.mi.48.100194.001205 7826005

[mmi14167-bib-0030] Carden, S.E. , Walker, G.T. , Honeycutt, J. , Lugo, K. , Pham, T. , Jacobson, A. *et al* (2017) Pseudogenization of the secreted effector gene ssei confers rapid systemic dissemination of *S.* Typhimurium ST313 within migratory dendritic cells. Cell Host & Microbe, 21, 182–194. 28182950 10.1016/j.chom.2017.01.009 PMC5325708

[mmi14167-bib-0031] Cardinale, C.J. , Washburn, R.S. , Tadigotla, V.R. , Brown, L.M. , Gottesman, M.E. and Nudler, E. (2008) Termination factor Rho and its cofactors NusA and NusG silence foreign DNA in *E. coli* . Science, 320, 935–938. 18487194 10.1126/science.1152763 PMC4059013

[mmi14167-bib-0032] Casadesús, J. (2016) Bacterial DNA methylation and methylomes. Advances in Experimental Medicine and Biology, 945, 35–61. 27826834 10.1007/978-3-319-43624-1_3

[mmi14167-bib-0033] Casjens, S. (2003) Prophages and bacterial genomics: what have we learned so far? Molecular Microbiology, 49, 277–300. 12886937 10.1046/j.1365-2958.2003.03580.x

[mmi14167-bib-0034] Casjens, S.R. and Grose, J.H. (2016) Contributions of P2‐ and P22‐like prophages to understanding the enormous diversity and abundance of tailed bacteriophages. Virology, 496, 255–276. 27372181 10.1016/j.virol.2016.05.022 PMC4969182

[mmi14167-bib-0035] Cenens, W. , Makumi, A. , Govers, S.K. , Lavigne, R. and Aertsen, A. (2015) Viral transmission dynamics at single‐cell resolution reveal transiently immune subpopulations caused by a carrier state association. PLoS Genetics, 11, e1005770. 26720743 10.1371/journal.pgen.1005770 PMC4697819

[mmi14167-bib-0036] Cenens, W. , Makumi, A. , Mebrhatu, M.T. , Lavigne, R. and Aertsen, A. (2013a) Phage‐host interactions during pseudolysogeny: lessons from the Pid/dgo interaction. Bacteriophage, 3, e25029. 23819109 10.4161/bact.25029 PMC3694060

[mmi14167-bib-0037] Cenens, W. , Mebrhatu, M.T. , Makumi, A. , Ceyssens, P.‐J. , Lavigne, R. , Van Houdt, R. *et al* (2013b) Expression of a novel P22 ORFan gene reveals the phage carrier state in Salmonella Typhimurium. PLoS Genetics, 9, e1003269. 23483857 10.1371/journal.pgen.1003269 PMC3573128

[mmi14167-bib-0038] Chen, Y. , Golding, I. , Sawai, S. , Guo, L. and Cox, E.C. (2005) Population fitness and the regulation of *Escherichia coli* genes by bacterial viruses. PLoS Biology, 3, e229. 15984911 10.1371/journal.pbio.0030229 PMC1151598

[mmi14167-bib-0039] Clokie M.R.J. and Kropinski A.M. (Eds.) (2009) Bacteriophages: Methods and Protocols. New York: Humana Press.

[mmi14167-bib-0040] Colgan, A.M. , Kröger, C. , Diard, M. , Hardt, W.‐D. , Puente, J.L. , Sivasankaran, S.K. *et al* (2016) The impact of 18 ancestral and horizontally‐acquired regulatory proteins upon the transcriptome and sRNA landscape of *Salmonella enterica* serovar Typhimurium. PLoS Genetics, 12, e1006258. 27564394 10.1371/journal.pgen.1006258 PMC5001712

[mmi14167-bib-0041] Coombes, B.K. , Wickham, M.E. , Brown, N.F. , Lemire, S. , Bossi, L. , Hsiao, W.W.L. *et al* (2005) Genetic and molecular analysis of GogB, a phage‐encoded type III‐secreted substrate in *Salmonella enterica* serovar typhimurium with autonomous expression from its associated phage. Journal of Molecular Biology, 348, 817–830. 15843015 10.1016/j.jmb.2005.03.024

[mmi14167-bib-0042] Cota, I. , Sánchez‐Romero, M.A. , Hernández, S.B. , Pucciarelli, M.G. , García‐del Portillo, F. and Casadesús, J. (2015) Epigenetic control of *Salmonella enterica* O‐antigen chain length: a tradeoff between virulence and bacteriophage resistance. PLoS Genetics, 11(11), e1005667. 26583926 10.1371/journal.pgen.1005667 PMC4652898

[mmi14167-bib-0043] Cresawn, S.G. , Bogel, M. , Day, N. , Jacobs‐Sera, D. , Hendrix, R.W. and Hatfull, G.F. (2011) Phamerator: a bioinformatic tool for comparative bacteriophage genomics. BMC Bioinformatics, 12, 395. 21991981 10.1186/1471-2105-12-395 PMC3233612

[mmi14167-bib-0044] D’Alessandro, B. , Pérez Escanda, V. , Balestrazzi, L. , Iriarte, A. , Pickard, D. , Yim, L. *et al* (2018) A novel prophage identified in strains from *Salmonella enterica *serovar enteritidis is a phylogenetic signature of the lineage ST‐1974. Microbial Genomics, 4. 10.1099/mgen.0.000161 PMC5885013 29509137

[mmi14167-bib-0045] D’Ari, R. and Casadesús, J. (1998) Underground metabolism. BioEssays: News and Reviews in Molecular, Cellular and Developmental Biology, 20, 181–186. 10.1002/(SICI)1521-1878(199802)20:2<181::AID-BIES10>3.0.CO;2-0 9631663

[mmi14167-bib-0046] Darwin, K.H. and Miller, V.L. (2000) The putative invasion protein chaperone SicA acts together with InvF to activate the expression of *Salmonella typhimurium* virulence genes. Molecular Microbiology, 35, 949–960. 10692170 10.1046/j.1365-2958.2000.01772.x

[mmi14167-bib-0047] Davies, E.V. , Winstanley, C. , Fothergill, J.L. and James, C.E. (2016) The role of temperate bacteriophages in bacterial infection. FEMS Microbiology Letters, 363, fnw015. 26825679 10.1093/femsle/fnw015

[mmi14167-bib-0048] Davies, M.R. , Broadbent, S.E. , Harris, S.R. , Thomson, N.R. and van der Woude, M.W. (2013) Horizontally acquired glycosyltransferase operons drive *Salmonellae* lipopolysaccharide diversity. PLoS Genetics, 9, e1003568. 23818865 10.1371/journal.pgen.1003568 PMC3688519

[mmi14167-bib-0049] De Paepe, M. , Hutinet, G. , Son, O. , Amarir‐Bouhram, J. , Schbath, S. and Petit, M.‐A. (2014) Temperate phages acquire DNA from defective prophages by relaxed homologous recombination: the role of Rad52‐like recombinases. PLoS Genetics, 10, e1004181. 24603854 10.1371/journal.pgen.1004181 PMC3945230

[mmi14167-bib-0050] Dearborn, A.D. and Dokland, T. (2012) Mobilization of pathogenicity islands by *Staphylococcus aureus* strain Newman bacteriophages. Bacteriophage, 2, 70–78. 23050217 10.4161/bact.20632 PMC3442828

[mmi14167-bib-0051] Diard, M. , Bakkeren, E. , Cornuault, J.K. , Moor, K. , Hausmann, A. , Sellin, M.E. *et al* (2017) Inflammation boosts bacteriophage transfer between *Salmonella* spp. Science, 355, 1211–1215. 28302859 10.1126/science.aaf8451

[mmi14167-bib-0052] Dorman, C.J. (2007) H‐NS, the genome sentinel. Nature Reviews Microbiology, 5, 157–161. 17191074 10.1038/nrmicro1598

[mmi14167-bib-0053] Duerr, C.U. , Zenk, S.F. , Chassin, C. , Pott, J. , Gütle, D. , Hensel, M. *et al* (2009) O‐antigen delays lipopolysaccharide recognition and impairs antibacterial host defense in murine intestinal epithelial cells. PLoS Pathogens, 5, e1000567. 19730692 10.1371/journal.ppat.1000567 PMC2729928

[mmi14167-bib-0054] Ehrbar, K. and Hardt, W.‐D. (2005) Bacteriophage‐encoded type III effectors in *Salmonella enterica* subspecies 1 serovar Typhimurium. Infection, Genetics and Evolution: Journal of Molecular Epidemiology and Evolutionary Genetics in Infectious Diseases, 5, 1–9. 15567133 10.1016/j.meegid.2004.07.004

[mmi14167-bib-0055] Enault, F. , Briet, A. , Bouteille, L. , Roux, S. , Sullivan, M.B. and Petit, M.‐A. (2017) Phages rarely encode antibiotic resistance genes: a cautionary tale for virome analyses. The ISME Journal, 11, 237–247. 27326545 10.1038/ismej.2016.90 PMC5315482

[mmi14167-bib-0056] Evans, M.R. , Fink, R.C. , Vazquez‐Torres, A. , Porwollik, S. , Jones‐Carson, J. , McClelland, M. *et al* (2011) Analysis of the ArcA regulon in anaerobically grown *Salmonella enterica* sv. Typhimurium. BMC Microbiology, 11, 58. 21418628 10.1186/1471-2180-11-58 PMC3075218

[mmi14167-bib-0057] Feiner, R. , Argov, T. , Rabinovich, L. , Sigal, N. , Borovok, I. and Herskovits, A.A. (2015) A new perspective on lysogeny: prophages as active regulatory switches of bacteria. Nature Reviews Microbiology, 13, 641–650. 26373372 10.1038/nrmicro3527

[mmi14167-bib-0058] Feng, X. , Walthers, D. , Oropeza, R. and Kenney, L.J. (2004) The response regulator SsrB activates transcription and binds to a region overlapping OmpR binding sites at *Salmonella *pathogenicity island 2. Molecular Microbiology, 54, 823–835. 15491370 10.1111/j.1365-2958.2004.04317.x

[mmi14167-bib-0059] Figueroa‐Bossi, N. and Bossi, L. (1999) Inducible prophages contribute to *Salmonella *virulence in mice. Molecular Microbiology, 33, 167–176. 10411733 10.1046/j.1365-2958.1999.01461.x

[mmi14167-bib-0060] Figueroa‐Bossi, N. and Bossi, L. (2004) Resuscitation of a defective prophage in *Salmonella* cocultures. Journal of Bacteriology, 186, 4038–4041. 15175320 10.1128/JB.186.12.4038-4041.2004 PMC419927

[mmi14167-bib-0061] Figueroa‐Bossi, N. , Coissac, E. , Netter, P. and Bossi, L. (1997) Unsuspected prophage‐like elements in *Salmonella *typhimurium. Molecular Microbiology, 25, 161–173. 11902718 10.1046/j.1365-2958.1997.4451807.x

[mmi14167-bib-0062] Figueroa‐Bossi, N. , Uzzau, S. , Maloriol, D. and Bossi, L. (2001) Variable assortment of prophages provides a transferable repertoire of pathogenic determinants in *Salmonella* . Molecular Microbiology, 39, 260–271. 11136448 10.1046/j.1365-2958.2001.02234.x

[mmi14167-bib-0063] Fink, R.C. , Evans, M.R. , Porwollik, S. , Vazquez‐Torres, A. , Jones‐Carson, J. , Troxell, B. *et al* (2007) FNR is a global regulator of virulence and anaerobic metabolism in *Salmonella enterica* Serovar Typhimurium (ATCC 14028s). Journal of Bacteriology, 189, 2262–2273. 17220229 10.1128/JB.00726-06 PMC1899381

[mmi14167-bib-0064] Fortier, L.‐C. and Sekulovic, O. (2013) Importance of prophages to evolution and virulence of bacterial pathogens. Virulence, 4, 354–365. 23611873 10.4161/viru.24498 PMC3714127

[mmi14167-bib-0065] García‐Pastor, L. , Puerta‐Fernández, E. and Casadesús, J. (2018) Bistability and phase variation in *Salmonella enterica* . Biochimica et Biophysica Acta (BBA) – Gene Regulatory Mechanisms. 10.1016/j.bbagrm.2018.01.003 29369799

[mmi14167-bib-0066] Gilcrease, E.B. and Casjens, S.R. (2018) The genome sequence of *Escherichia coli *tailed phage D6 and the diversity of Enterobacteriales circular plasmid prophages. Virology, 515, 203–214. 29304472 10.1016/j.virol.2017.12.019 PMC5800970

[mmi14167-bib-0067] Hammarlöf, D.L. , Kröger, C. , Owen, S.V. , Canals, R. , Lacharme‐Lora, L. , Wenner, N. *et al* (2018) Role of a single noncoding nucleotide in the evolution of an epidemic African clade of *Salmonella* . Proceedings of the National Academy of Sciences, 115, E2614–E2623. 10.1073/pnas.1714718115 PMC5856525 29487214

[mmi14167-bib-0068] Hatfull, G.F. (2015) Dark matter of the biosphere: the amazing world of bacteriophage diversity. Journal of Virology, 89, 8107–8110. 26018169 10.1128/JVI.01340-15 PMC4524254

[mmi14167-bib-0069] Hatfull, G.F. , Jacobs‐Sera, D. , Lawrence, J.G. , Pope, W.H. , Russell, D.A. , Ko, C.‐C. *et al* (2010) Comparative genomic analysis of 60 mycobacteriophage genomes: genome clustering, gene acquisition, and gene size. Journal of Molecular Biology, 397, 119–143. 20064525 10.1016/j.jmb.2010.01.011 PMC2830324

[mmi14167-bib-0070] Hernández, S.B. , Cota, I. , Ducret, A. , Aussel, L. and Casadesús, J. (2012) Adaptation and preadaptation of *Salmonella enterica* to Bile. PLoS Genetics, 8, e1002459. 22275872 10.1371/journal.pgen.1002459 PMC3261920

[mmi14167-bib-0071] Herrero‐Fresno, A. , Espinel, I. C. , Spiegelhauer, M. R. , Guerra, P. R. , Andersen, K. W. , and Olsen, J. E. (2018) The homolog of the gene bstA of the BTP1 Phage from Salmonella enterica Serovar Typhimurium ST313 is an antivirulence gene in Salmonella enterica Serovar Dublin. Infection and Immunity, 86. 10.1128/IAI.00784-17 PMC5736821 29109173

[mmi14167-bib-0072] Herrero‐Fresno, A. , Wallrodt, I. , Leekitcharoenphon, P. , Olsen, J.E. , Aarestrup, F.M. and Hendriksen, R.S. (2014) The role of the st313‐td Gene in virulence of *Salmonella* Typhimurium ST313. PLoS One, 9, e84566. 24404174 10.1371/journal.pone.0084566 PMC3880295

[mmi14167-bib-0073] Hershko‐Shalev, T. , Odenheimer‐Bergman, A. , Elgrably‐Weiss, M. , Ben‐Zvi, T. , Govindarajan, S. , Seri, H. et al. (2016) Gifsy‐1 prophage IsrK with dual function as small and messenger RNA modulates vital bacterial machineries. PLoS Genetics, 12, e1005975. 27057757 10.1371/journal.pgen.1005975 PMC4825925

[mmi14167-bib-0074] Ho, T.D. and Slauch, J.M. (2001) Characterization of *grvA*, an antivirulence gene on the gifsy‐2 phage in *Salmonella enterica* serovar typhimurium. Journal of Bacteriology, 183, 611–620. 11133955 10.1128/JB.183.2.611-620.2001 PMC94917

[mmi14167-bib-0075] Hoffmann, M. , Zhao, S. , Pettengill, J. , Luo, Y. , Monday, S.R. , Abbott, J. *et al* (2014) Comparative genomic analysis and virulence differences in closely related *Salmonella enterica* serotype heidelberg isolates from humans, retail meats, and animals. Genome Biology and Evolution, 6, 1046–1068. 24732280 10.1093/gbe/evu079 PMC4040988

[mmi14167-bib-0076] Jaslow, S.L. , Gibbs, K.D. , Fricke, W.F. , Wang, L. , Pittman, K.J. , Mammel, M.K. *et al* (2018) Salmonella activation of STAT3 signaling by SarA effector promotes intracellular replication and production of IL‐10. Cell Reports, 23, 3525–3536. 29924996 10.1016/j.celrep.2018.05.072 PMC6314477

[mmi14167-bib-0077] Kamal, F. and Dennis, J.J. (2015) Burkholderia cepacia complex phage‐antibiotic synergy (PAS): antibiotics stimulate lytic phage activity. Applied and Environmental Microbiology, 81, 1132–1138. 25452284 10.1128/AEM.02850-14 PMC4292504

[mmi14167-bib-0078] Kaur, J. and Jain, S.K. (2012) Role of antigens and virulence factors of *Salmonella enterica *serovar Typhi in its pathogenesis. Microbiological Research, 167, 199–210. 21945101 10.1016/j.micres.2011.08.001

[mmi14167-bib-0079] Kim, M. and Ryu, S. (2013) Antirepression system associated with the life cycle switch in the temperate podoviridae phage SPC32H. Journal of Virolog, 87, 11775–11786. 10.1128/JVI.02173-13 PMC3807360 23986584

[mmi14167-bib-0080] Kim, S. , Ryu, K. , Biswas, D. and Ahn, J. (2014) Survival, prophage induction, and invasive properties of lysogenic *Salmonella* Typhimurium exposed to simulated gastrointestinal conditions. Archives of Microbiology, 196, 655–659. 24929817 10.1007/s00203-014-1005-z

[mmi14167-bib-0081] Kintz, E. , Davies, M.R. , Hammarlöf, D.L. , Canals, R. , Hinton, J.C.D. and van der Woude, M.W. (2015) A BTP1 prophage gene present in invasive non‐typhoidal *Salmonella *determines composition and length of the O‐antigen of the lipopolysaccharide. Molecular Microbiology, 96, 263–275. 25586744 10.1111/mmi.12933 PMC4413052

[mmi14167-bib-0082] Kraushaar, B. , Hammerl, J.A. , Kienöl, M. , Heinig, M.L. , Sperling, N. , Dinh Thanh, M. *et al* (2017) Acquisition of virulence factors in livestock‐associated MRSA: lysogenic conversion of CC398 strains by virulence gene‐containing phages. Scientific Reports, 7, 2004. 28515479 10.1038/s41598-017-02175-4 PMC5435737

[mmi14167-bib-0083] Kröger, C. , Colgan, A. , Srikumar, S. , Händler, K. , Sivasankaran, S.K. , Hammarlöf, D.L. *et al* (2013) An infection‐relevant transcriptomic compendium for *Salmonella enterica* Serovar Typhimurium. Cell Host & Microbe, 14, 683–695. 24331466 10.1016/j.chom.2013.11.010

[mmi14167-bib-0084] Kropinski, A.M. , Kovalyova, I.V. , Billington, S.J. , Patrick, A.N. , Butts, B.D. , Guichard, J.A. *et al* (2007) The genome of epsilon15, a serotype‐converting, Group E1 *Salmonella enterica*‐specific bacteriophage. Virology, 369, 234–244. 17825342 10.1016/j.virol.2007.07.027 PMC2698709

[mmi14167-bib-0085] Lemire, S. , Figueroa‐Bossi, N. and Bossi, L. (2011) Bacteriophage crosstalk: coordination of prophage induction by trans‐acting antirepressors. PLoS Genetics, 7, e1002149. 21731505 10.1371/journal.pgen.1002149 PMC3121763

[mmi14167-bib-0086] Lévi‐Meyrueis, C. , Monteil, V. , Sismeiro, O. , Dillies, M.‐A. , Kolb, A. , Monot, M. *et al* (2015) Repressor activity of the RpoS/σS‐dependent RNA polymerase requires DNA binding. Nucleic Acids Research, 43, 1456–1468. 25578965 10.1093/nar/gku1379 PMC4330354

[mmi14167-bib-0087] Levine, M. , Truesdell, S. , Ramakrishnan, T. and Bronson, M.J. (1975) Dual control of lysogeny by bacteriophage P22: an antirepressor locus and its controlling elements. Journal of Molecular Biology, 91, 421–438. 1097696 10.1016/0022-2836(75)90270-3

[mmi14167-bib-0088] Lewis, M. (2011) A tale of two repressors – a historical perspective. Journal of Molecular Biology, 409, 14–27. 21392509 10.1016/j.jmb.2011.02.023 PMC3104267

[mmi14167-bib-0089] Lopes, A. , Amarir‐Bouhram, J. , Faure, G. , Petit, M.‐A. and Guerois, R. (2010) Detection of novel recombinases in bacteriophage genomes unveils Rad52, Rad51 and Gp2.5 remote homologs. Nucleic Acids Research, 38, 3952–3962. 20194117 10.1093/nar/gkq096 PMC2896510

[mmi14167-bib-0090] Lucchini, S. , Rowley, G. , Goldberg, M.D. , Hurd, D. , Harrison, M. and Hinton, J.C.D. (2006) H‐NS mediates the silencing of laterally acquired genes in bacteria. PLoS Pathogens, 2, e81. 16933988 10.1371/journal.ppat.0020081 PMC1550270

[mmi14167-bib-0091] Menouni, R. , Hutinet, G. , Petit, M.‐A. and Ansaldi, M. (2015) Bacterial genome remodeling through bacteriophage recombination. FEMS Microbiology Letters, 362, 1–10. 10.1093/femsle/fnu022 25790500

[mmi14167-bib-0092] Mirold, S. , Rabsch, W. , Tschäpe, H. and Hardt, W.D. (2001) Transfer of the *Salmonella* type III effector sopE between unrelated phage families. Journal of Molecular Biology, 312, 7–16. 11545581 10.1006/jmbi.2001.4950

[mmi14167-bib-0093] Nadeem, A. and Wahl, L.M. (2017) Prophage as a genetic reservoir: promoting diversity and driving innovation in the host community. Evolution: International Journal of Organic Evolution, 71, 2080–2089. 28590013 10.1111/evo.13287

[mmi14167-bib-0094] Navarre, W.W. , Halsey, T.A. , Walthers, D. , Frye, J. , McClelland, M. , Potter, J.L. *et al* (2005) Co‐regulation of Salmonella enterica genes required for virulence and resistance to antimicrobial peptides by SlyA and PhoP/PhoQ. Molecular Microbiology, 56, 492–508. 15813739 10.1111/j.1365-2958.2005.04553.x

[mmi14167-bib-0095] Navarre, W.W. , McClelland, M. , Libby, S.J. and Fang, F.C. (2007) Silencing of xenogeneic DNA by H‐NS – facilitation of lateral gene transfer in bacteria by a defense system that recognizes foreign DNA. Genes & Development, 21, 1456–1471. 17575047 10.1101/gad.1543107

[mmi14167-bib-0096] Navarre, W.W. , Porwollik, S. , Wang, Y. , McClelland, M. , Rosen, H. , Libby, S.J. *et al* (2006) Selective silencing of foreign DNA with low GC content by the H‐NS protein in *Salmonella* . Science, 313, 236–238. 16763111 10.1126/science.1128794

[mmi14167-bib-0097] Nedialkova, L.P. , Sidstedt, M. , Koeppel, M.B. , Spriewald, S. , Ring, D. , Gerlach, R.G. *et al* (2015) Temperate phages promote colicin‐dependent fitness of *Salmonella enterica* serovar Typhimurium. Environmental Microbiology, 18, 1591–1603. 26439675 10.1111/1462-2920.13077

[mmi14167-bib-0098] Oliveira, P.H. , Touchon, M. , Cury, J. and Rocha, E.P.C. (2017) The chromosomal organization of horizontal gene transfer in bacteria. Nature Communications, 8, 841. 10.1038/s41467-017-00808-w PMC5635113 29018197

[mmi14167-bib-0099] Owen, S.V. , Wenner, N. , Canals, R. , Makumi, A. , Hammarlöf, D.L. , Gordon, M.A. *et al* (2017) Characterization of the prophage repertoire of African *Salmonella* Typhimurium ST313 reveals high levels of spontaneous induction of novel phage BTP1. Frontiers in Microbiology, 8, 235. 28280485 10.3389/fmicb.2017.00235 PMC5322425

[mmi14167-bib-0100] Pilar, A.V.C. , Reid‐Yu, S.A. , Cooper, C.A. , Mulder, D.T. and Coombes, B.K. (2012) GogB is an anti‐inflammatory effector that limits tissue damage during *Salmonella *infection through interaction with human FBXO22 and Skp1. PLoS Pathogens, 8, e1002773. 22761574 10.1371/journal.ppat.1002773 PMC3386239

[mmi14167-bib-0101] Porwollik, S. , Wong, R.M.‐Y. and McClelland, M. (2002) Evolutionary genomics of *Salmonella*: gene acquisitions revealed by microarray analysis. Proceedings of the National Academy of Sciences, 99, 8956–8961. 10.1073/pnas.122153699 PMC124405 12072558

[mmi14167-bib-0102] Rabinovich, L. , Sigal, N. , Borovok, I. , Nir‐Paz, R. and Herskovits, A.A. (2012) Prophage excision activates *Listeria* competence genes that promote phagosomal escape and virulence. Cell, 150, 792–802. 22901809 10.1016/j.cell.2012.06.036

[mmi14167-bib-0103] Ranade, K. and Poteete, A.R. (1993) Superinfection exclusion (*sieB*) genes of bacteriophages P22 and lambda. Journal of Bacteriology, 175, 4712–4718. 8335629 10.1128/jb.175.15.4712-4718.1993 PMC204922

[mmi14167-bib-0104] Rinke, C. , Schwientek, P. , Sczyrba, A. , Ivanova, N.N. , Anderson, I.J. , Cheng, J.‐F. *et al* (2013) Insights into the phylogeny and coding potential of microbial dark matter. Nature, 499, 431–437. 23851394 10.1038/nature12352

[mmi14167-bib-0105] Roux, S. , Enault, F. , Hurwitz, B.L. and Sullivan, M.B. (2015a) VirSorter: mining viral signal from microbial genomic data. PeerJournal, 3, e985. 10.7717/peerj.985 PMC4451026 26038737

[mmi14167-bib-0106] Roux, S. , Hallam, S.J. , Woyke, T. and Sullivan, M.B. (2015b) Viral dark matter and virus–host interactions resolved from publicly available microbial genomes. eLife, 4. 10.7554/eLife.08490 PMC4533152 26200428

[mmi14167-bib-0107] Sauer, R.T. , Pan, J. , Hopper, P. , Hehir, K. , Brown, J. and Poteete, A.R. (1981) Primary structure of the phage P22 repressor and its gene c2. Biochemistry, 20, 3591–3598. 7260059 10.1021/bi00515a044

[mmi14167-bib-0108] Shah, J. , Desai, P.T. and Weimer, B.C. (2014) Genetic mechanisms underlying the pathogenicity of cold‐stressed *Salmonella enterica *Serovar Typhimurium in cultured intestinal epithelial cells. Applied and Environmental Microbiology, 80, 6943–6953. 25192993 10.1128/AEM.01994-14 PMC4249018

[mmi14167-bib-0109] Shearwin, K.E. , Brumby, A.M. and Egan, J.B. (1998) The tum protein of coliphage 186 is an antirepressor. Journal of Biological Chemistry, 273, 5708–5715. 9488703 10.1074/jbc.273.10.5708

[mmi14167-bib-0110] Slauch, J.M. , Lee, A.A. , Mahan, M.J. and Mekalanos, J.J. (1996) Molecular characterization of the *oafA* locus responsible for acetylation of *Salmonella *typhimurium O‐antigen: *oafA* is a member of a family of integral membrane trans‐acylases. Journal of Bacteriology, 178, 5904–5909. 8830685 10.1128/jb.178.20.5904-5909.1996 PMC178445

[mmi14167-bib-0111] Sun, Q. , Knirel, Y.A. , Wang, J. , Luo, X. , Senchenkova, S.N. , Lan, R. *et al* (2014) Serotype‐converting bacteriophage SfII encodes an acyltransferase protein that mediates 6‐O‐acetylation of GlcNAc in Shigella flexneri O‐antigens, conferring on the host a novel O‐antigen epitope. Journal of Bacteriology, 196, 3656–3666. 25112477 10.1128/JB.02009-14 PMC4187690

[mmi14167-bib-0112] Susskind, M.M. and Botstein, D. (1980) Superinfection exclusion by lambda prophage in lysogens of *Salmonella* typhimurium. Virology, 100, 212–216. 6444259 10.1016/0042-6822(80)90571-1

[mmi14167-bib-0113] Susskind, M.M. , Botstein, D. and Wright, A. (1974) Superinfection exclusion by P22 prophage in lysogens of *Salmonella* typhimurium. III. Failure of superinfecting phage DNA to enter *sieA*+ lysogens. Virology, 62, 350–366. 4610992 10.1016/0042-6822(74)90398-5

[mmi14167-bib-0114] Suttle, C.A. (2007) Marine viruses – major players in the global ecosystem. Nature Reviews Microbiology, 5, 801–812. 17853907 10.1038/nrmicro1750

[mmi14167-bib-0115] Tomljenovic‐Berube, A.M. , Henriksbo, B. , Porwollik, S. , Cooper, C.A. , Tuinema, B.R. , McClelland, M. *et al* (2013) Mapping and regulation of genes within *Salmonella* pathogenicity island 12 that contribute to in vivo fitness of Salmonella enterica Serovar Typhimurium. Infection and Immunity, 81, 2394–2404. 23630960 10.1128/IAI.00067-13 PMC3697593

[mmi14167-bib-0116] Touchon, M. and Rocha, E.P.C. (2010) The small, slow and specialized CRISPR and anti‐CRISPR of *Escherichia* and *Salmonella* . PloS One, 5, e11126. 20559554 10.1371/journal.pone.0011126 PMC2886076

[mmi14167-bib-0117] Tucker, S.C. and Galán, J.E. (2000) Complex function for SicA, a *Salmonella enterica* serovar typhimurium type III secretion‐associated chaperone. Journal of Bacteriology, 182, 2262–2268. 10735870 10.1128/jb.182.8.2262-2268.2000 PMC111276

[mmi14167-bib-0118] Vivero, A. , Baños, R.C. , Mariscotti, J.F. , Oliveros, J.C. , García‐del Portillo, F. , Juárez, A. *et al* (2008) Modulation of horizontally acquired genes by the Hha‐YdgT proteins in *Salmonella enterica* serovar Typhimurium. Journal of Bacteriology, 190, 1152–1156. 18039769 10.1128/JB.01206-07 PMC2223552

[mmi14167-bib-0119] Vonaesch, P. , Sellin, M.E. , Cardini, S. , Singh, V. , Barthel, M. and Hardt, W.‐D. (2014) The *Salmonella* Typhimurium effector protein SopE transiently localizes to the early SCV and contributes to intracellular replication. Cellular Microbiology, 16, 1723–1735. 25052734 10.1111/cmi.12333

[mmi14167-bib-0120] Wernicki, A. , Nowaczek, A. and Urban‐Chmiel, R. (2017) Bacteriophage therapy to combat bacterial infections in poultry. Virology Journal, 14, 179. 28915819 10.1186/s12985-017-0849-7 PMC5602926

[mmi14167-bib-0121] Whipple, F.W. , Hou, E.F. and Hochschild, A. (1998) Amino acid‐amino acid contacts at the cooperativity interface of the bacteriophage lambda and P22 repressors. Genes & Development, 12, 2791–2802. 9732276 10.1101/gad.12.17.2791 PMC317150

[mmi14167-bib-0122] Worley, M.J. , Ching, K.H. and Heffron, F. (2000) *Salmonella* SsrB activates a global regulon of horizontally acquired genes. Molecular Microbiology, 36, 749–761. 10844662 10.1046/j.1365-2958.2000.01902.x

[mmi14167-bib-0123] Yang, Z. , Soderholm, A. , Lung, T.W.F. , Giogha, C. , Hill, M.M. , Brown, N.F. *et al* (2015) SseK3 is a *Salmonella* effector that binds TRIM32 and modulates the host’s NF‐κB signalling activity. PLoS One, 10, e0138529. 26394407 10.1371/journal.pone.0138529 PMC4579058

[mmi14167-bib-0124] Yoon, H. , Ansong, C. , Adkins, J.N. and Heffron, F. (2011) Discovery of *Salmonella* virulence factors translocated via outer membrane vesicles to murine macrophages. Infection and Immunity, 79, 2182–2192. 21464085 10.1128/IAI.01277-10 PMC3125828

[mmi14167-bib-0125] Zinder, N.D. and Lederberg, J. (1952) Genetic exchange in *Salmonella* . Journal of Bacteriology, 64, 679–699. 12999698 10.1128/jb.64.5.679-699.1952 PMC169409

[mmi14167-bib-0126] Zou, Q.‐H. , Li, Q.‐H. , Zhu, H.‐Y. , Feng, Y. , Li, Y.‐G. , Johnston, R.N. *et al* (2010) SPC‐P1: a pathogenicity‐associated prophage of *Salmonella *paratyphi C. BMC Genomics, 11, 729. 21192789 10.1186/1471-2164-11-729 PMC3022927

